# Dating the key structures of the Guge ancient city through integrated ^14^C and literary research

**DOI:** 10.1371/journal.pone.0353853

**Published:** 2026-09-30

**Authors:** Xiaoli He, Changqing Fu, Jiaming Niu, Shargan Wangdue, Zhijian Cao, Jianlin Zhang

**Affiliations:** 1 Department of Art and Design, Guangzhou Maritime University, Guangzhou, Guangdong, China; 2 Visual Culture Research Center, Guangzhou Academy of Fine Arts, Guangzhou, Guangdong, China; 3 Tibetan Institute of the Preservation of Cultural Relics, Lhasa, China; 4 Department of Cultural Heritage Protection and anagement, Guangzhou Academy of Fine Arts, Guangzhou, Guangdong, China; 5 Shaanxi Academy of Archaeology, Xi'an, China; Ain Shams University, EGYPT

## Abstract

This study integrates AMS ^14^C dating of 12 plant specimens, Bayesian modeling, and Tibetan texts to establish the first high-resolution construction chronology for key structures of the Guge ancient city in western Tibet. To ensure chronological precision and mitigate the old-wood effect, sampling prioritized short-lived annual plants and the outermost growth layers of small-diameter timber. Radiocarbon data were processed using the IntCal20 calibration curve and constrained by independent historical *termini ante quos* derived from mural inscriptions and prayer texts. The findings delineate three primary developmental phases: an Early Construction Phase (mid-to-late 15th century) encompassing the VIF27 complex, dated to 1457–1471 CE; a White Temple Phase (mid-16th century) focused on Buddha statues, which ended between 1556 and 1560 CE; and a Late Defensive Phase (mid-17th to late 18th century) involving the reinforcement of Watchtower VIII D2 (1661–1813 CE), possibly in response to regional conflicts. Notably, the use of herbaceous samples successfully resolved the 16th-century calibration plateau ambiguities, aligning the production closely with the reign of King Jig rten dbang phyug. This systematic framework provides a robust empirical foundation for understanding the architectural evolution and socio-political transformations of the Guge Kingdom.

## 1. Introduction

The Guge Kingdom, established in the 10th century in Ngari, Western Tibet (Fig 1), represents a pivotal period in the resurgence of Tibetan Buddhism following the collapse of the Tibetan Empire. While the kingdom’s early foundations were widely distributed across the region, its late-period political and cultural center was concentrated at Tsaparang. This capital served as the seat of power during the later period of the kingdom, from approximately the 15th century until the dissolution of the kingdom around 1630 CE [1]. The Guge ancient city site (31°25’ N, 79°39’ E) is one of the most significant archaeological complexes in High Asia, containing over 1,400 structures, including palaces, caves, and key religious monuments like the Mandala Hall and the White Temple (Fig 2).

**Fig 1:**
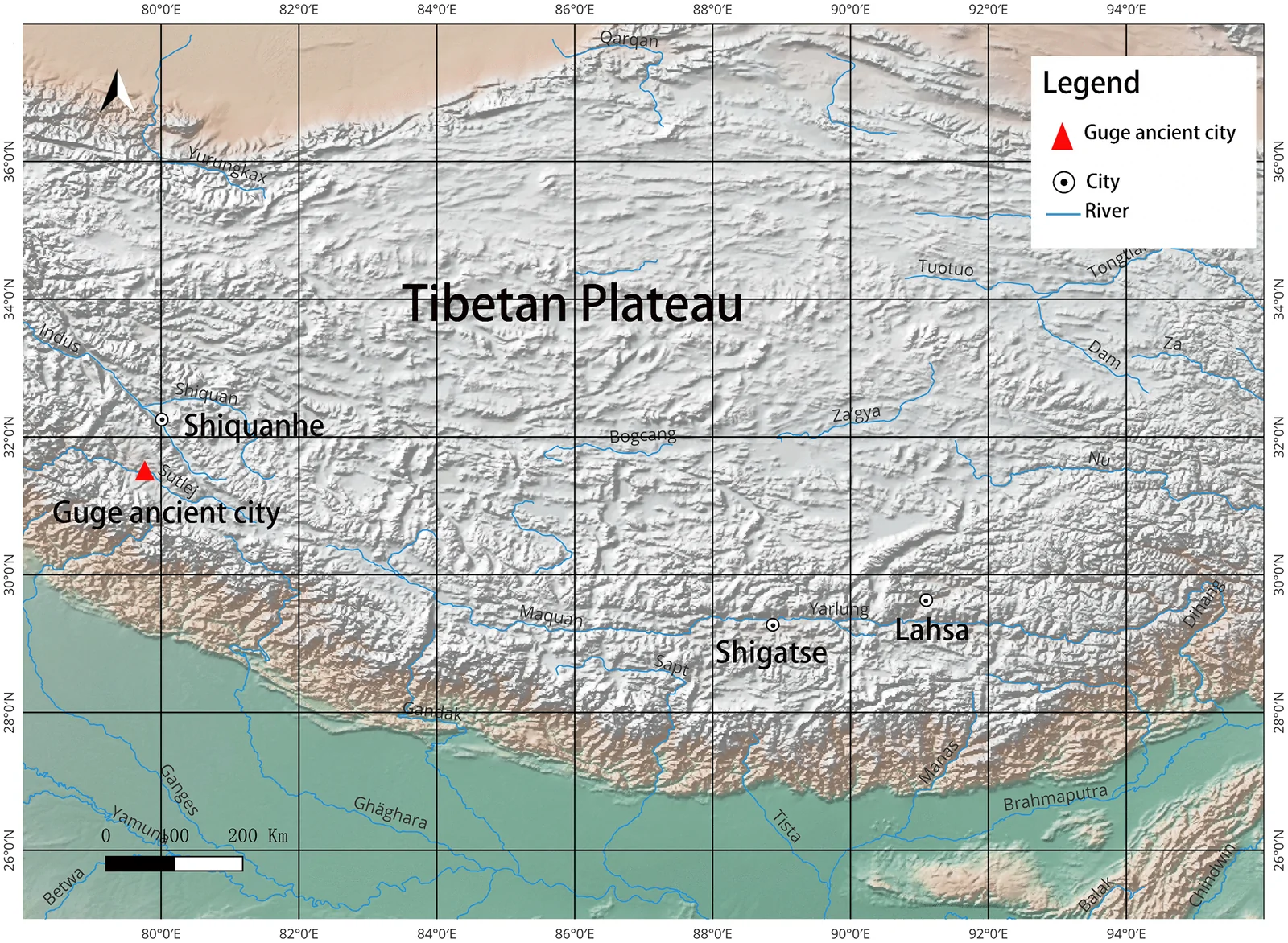
Geographical location and setting of the Guge Ancient City. Map by Xiaoli He with Natural Earth (free vector and raster map data@ naturalearthdata.com; CC BY 4.0).

**Fig 2:**
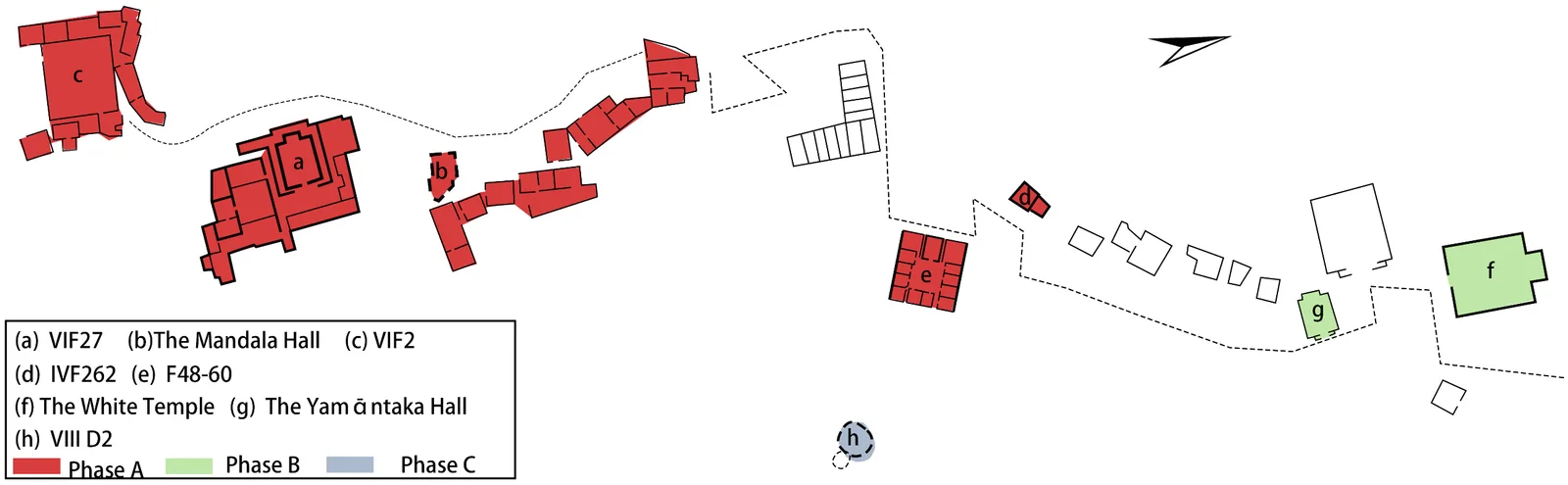
Site plan and construction phase diagram. Site plan by Xiaoli He, based on " *The site of the Ancient Guge Kingdom* " (CC BY 4.0).

Despite its historical prominence, establishing a high-resolution architectural chronology for the key structures of the Guge ancient city remains a formidable challenge. Due to the region’s geographic isolation, contemporary documentary records are exceptionally scarce. Consequently, traditional chronological assessments have relied heavily on stylistic and typological comparisons of murals and architectural forms. While these methods provide valuable insights, they often yield broad temporal ranges that lack the precision necessary to correlate specific construction phases with known historical reigns or the kingdom’s socio-political shifts. For example, Su Bai classified the F48–60 complex as a 10th–11th century *vihāra*, while the murals in the Mandala Hall have a *terminus post quem* (TPQ) of the early 15th century [2]. Such ambiguities underscore the need for independent scientific dating methods.

Radiocarbon dating (^14^C), pioneered by Willard Libby in the 1940s [3], is a cornerstone of archaeological chronology. With continuously improving precision, it is now widely applied in multidisciplinary research [4–7]. By combining radiometric data with independent priors (e.g., historical inscriptions or stratigraphic sequences), Bayesian modeling refines calibrated date ranges into precise posterior probability distributions. Within archaeology, integrating such data with other evidence enables the resolution of chronological questions, ranging from structures [8–12] and sites [13–15] to tombs [16–20], smelting sites [21–24], cave temples [25–27], artifacts [28–30], petroglyphs [31,32], and other unearthed artifacts [33–36]. While ^14^C dating has been successfully applied to various cultural heritage contexts globally, its systematic application to the architectural evolution of the Guge ancient city has remained limited.

The present study addresses this gap by presenting the first systematic AMS ^14^C chronological framework for the Guge ancient city. Using high-precision radiocarbon dating, we analyzed 12 plant specimens, including structural timber and annual herbaceous stalks. By synthesizing these results with a systematic evaluation of Tibetan historical texts and mural epigraphy, we aim to delineate the primary construction phases of the site. This integrated framework not only refines the temporal boundaries of Guge’s urban development but also provides a scientific basis for understanding the broader cultural and defensive transformations of Western Tibet.

## 2. Methods

### 2.1. Sample pretreatment, AMS measurement, and Bayesian modeling

Sample pretreatment and AMS measurement were performed at the High-Sensitivity AMS Center, Yamagata University, Japan (hereafter “the Center”). The treatment followed a standard acid-alkali-acid sequence:

Step 1: Surface cleaning. Ultrasonic cleaning and sequential solvent washes (acetone, chloroform-ether, acetone) were used to remove surface contaminants and lipids.

Step 2: Carbonate removal. The samples were heated in 1 M HCl (80 °C, 6 h) to eliminate carbonates and other inorganic carbon compounds that would bias the ^14^C age.

Step 3: Humic acid removal. The samples were heated in 0.1 M NaOH (80 °C, 5 min) to remove humic acids and alkali-soluble organic contaminants.

Step 4: Final acid rinse. A brief 1 M HCl rinse neutralized residual alkali and removed adsorbed atmospheric CO_2_.

After each step, samples were rinsed with deionized water until neutral. The cleaned and dried samples were combusted in a vacuum and converted to CO_2_, then reduced to graphite using H_2_ at 600 °C for 10 h. Graphitization employed an elemental analyzer–mass spectrometer–glass vacuum line system. ^14^C /^12^C ratios were measured by accelerator mass spectrometry (NEC 1.5SDH) at the Center.

Calibration and Bayesian modeling used OxCal v4.4.4 [37,38] and the IntCal20 curve [39]. Both 1σ and 2σ probability ranges are reported in Table 1.

**Table 1:** ^14^C ages and calibrated date ranges for samples from Guge Ancient City.

No	Lab ID	Sample ID	Type of sample	δ^13^C (‰)	^14^C Age (yr BP ± 1σ)	Calibrated Date (1σ, 68.3%)	Calibrated Date (2σ, 95.4%)
1	YU-19966	GAFA-2-No1	Wood	−24.28 ± 0.29	337 ± 20	1500CE (20.7%) 1526CE 1556CE (34.4%) 1600CE 1615CE (13.2%) 1632CE	1482CE (30.4%) 1530CE 1538CE (65.1%) 1636CE
2	YU-19967	GAFA-2-No2	Wood	−12.29 ± 0.25	309 ± 20	1522CE (54.9%) 1575CE 1625CE (13.4%) 1640CE	1504CE (74.7%) 1597CE 1616CE (20.7%) 1646CE
3	YU-19968	GAFA-2-No3	Wood	−24.49 ± 0.19	340 ± 20	1497CE (21.9%) 1524CE 1559CE (32.3%) 1601CE 1613CE (14.0%) 1631CE	1479CE (32.6%) 1530CE 1540CE (62.8%) 1635CE
4	YU-19969	GAFA-2-No4	Straw	−15.01 ± 0.22	314 ± 20	1521CE (55.4%) 1579CE 1624CE (12.8%) 1638CE	1499CE (75.7%) 1600CE 1615CE (19.7%) 1644CE
5	YU-19970	GAFA-2-No5	Wood	−25.58 ± 0.29	304 ± 20	1524CE (56.0%) 1572CE 1630CE (12.2%) 1641CE	1506CE (73.0%) 1595CE 1617CE (22.4%) 1649CE
6	YU-19971	GAFA-2-No6	Straw	−11.96 ± 0.30	332 ± 20	1502CE (18.7%) 1528CE 1551CE (36.1%) 1598CE 1616CE (13.5%) 1634CE	1490CE (95.4%) 1638CE
7	YU-19972	GAFA-2-No7	Wood	−24.44 ± 0.44	177 ± 20	1669CE (12.0%) 1683CE 1736CE (39.7%) 1781CE 1799CE (2.9%) 1802CE 1930CE (13.7%)	1661CE (19.2%) 1695CE 1725CE (55.9%) 1812CE 1916CE (20.4%)
8	YU-19973	GAFA-2-No8	Wood	−25.14 ± 0.31	171 ± 20	1670CE (12.3%) 1686CE 1733CE (37.6%) 1779CE 1799CE (5.0%) 1805CE 1928CE (13.3%) 1945CE	1662CE (18.2%) 1695CE 1725CE (53.2%) 1813CE 1839CE (0.8%) 1846CE 1852CE (2.4%) 1877CE 1916CE (20.9%)
9	YU-19974	GAFA-2-No9	Wood	−25.56 ± 0.25	398 ± 20	1450 CE (68.3%) 1487 CE	1445 CE (83.8%) 1506 CE 1595 CE (11.6%) 1618 CE
10	YU-19975	GAFA-2-No10	Wood	−24.75 ± 0.48	367 ± 20	1469 CE (41.5%) 1513 CE 1591 CE (26.8%) 1620 CE	1456 CE (55.2%) 1524 CE 1560 CE (0.5%) 1562 CE 1571 CE (39.8%) 1631 CE
11	YU-19976	GAFA-2-No11	Wood	−25.67 ± 0.23	373 ± 20	1460 CE (47.3%) 1505 CE 1596 CE (21.0%) 1617 CE	1455 CE (61.0%) 1523 CE 1575 CE (34.4%) 1625 CE
12	YU-19979	GAFA-2-No14	Wood	−27.65 ± 0.37	405 ± 20	1449 CE (68.3%) 1478 CE	1442 CE (88.9%) 1501 CE 1599 CE (6.6%) 1616 CE

*BP* refers to the number of years before the year 1950

### 2.2. Objective criteria for evaluating multimodal calibration ranges

A recurring challenge in radiocarbon dating of medieval and early modern sites is the multimodal nature of the IntCal20 calibration curve, which can yield multiple statistically equivalent calendar-age ranges for a single ^14^C determination. To avoid circular reasoning, we adopted the following objective, reproducible criteria:

When multiple samples from the same context are available, we use Bayesian modeling with independent prior information (stratigraphic order, historical *terminus ante quem* [TAQ] or TPQ) to resolve multimodality. For VIF27, the TAQ constraint (<1471 CE) was applied as a Boundary in a Sequence model (see Section 2.3.1), with the three wood samples grouped within a Phase and combined using OxCal’s *Combine* command to verify their statistical consistency. The unconstrained model retained a later peak (>1560 CE); the constrained model removed it entirely. This demonstrates how independent priors can objectively eliminate statistically possible but historically implausible ranges.

When only a single sample is available (e.g., IVF262), no Bayesian model can resolve multimodality without an independent prior. In such cases, we explicitly present all possible calibrated ranges, provide archaeological and historical arguments for preferring one range, and fully acknowledge alternative interpretations. We do not claim that the non-preferred range is impossible; we only argue that it is less consistent with the totality of evidence.

For post-1650 CE samples (e.g., Watchtower VIII D2), the calibration curve exhibits multiple fluctuations that produce several ranges with comparable probabilities. Here, we integrate all plausible ranges with documented historical events (conflicts, administrative changes) without statistically privileging any single range. The conclusion is presented as a range of possibilities (mid-17th to late 18th century) rather than a single date.

These criteria ensure that our chronological interpretations are transparent, reproducible, and not subject to subjective peak selection.

### 2.3. Historical and epigraphic constraints for Bayesian modeling

#### 2.3.1. Textual and epigraphic evidence for the construction date of the VIF27 complex.

Multiple historical sources indicate that the earliest monasteries were established during the reign of King Phun tshogs lde [40–42]. The royal palace complex encompassed not only palatial structures but also religious buildings dedicated to Buddhist rituals. Within the Buddha niche of VIF27, the remains of a Sumeru pedestal (also known as *Xumizuo* in Chinese) suggest it originally supported a monumental clay statue of Sakyamuni Buddha. Traces on the surrounding walls indicate the former placement of eight Arhat statues. Although the interior mural surfaces are now severely weathered, residual pigment fragments indicate that VIF27 and its surrounding structures served a significant religious function. Approximately 30 meters northeast of VIF27 lies the Mandala Hall, with the administrative and council area (VIF2) situated to the south. Therefore, the two structures are likely contemporary components of a single monastic complex. Su Bai’s analysis of Tibetan monastic layouts suggests the Mandala Hall was part of a broader complex centered on VIF27 [2].

The inscriptions accompanying a pair of donor portraits in the Mandala Hall murals provide pivotal chronological anchors, offering a potential temporal constraint for the structure’s construction. The inscription beneath the monastic figure reads: *lha rje btsun Shākya ‘od* (ལྷ་རྗེ་བཙུན་ཤཱ་ཀྱ་འོད་), identifying the “Divine Venerable Shākya ‘od,” while the text below the lay figure identifies him as *drung* Blo bzang (གྲོང་བློ་བཟང་). Historical records indicate that Phun tshogs lde took monastic vows in Kha char in 1449 CE and was given the religious name Shākya ‘od [43]. Therefore, the monastic figure is interpreted as the former Guge king Phun tshogs lde. The Tibetan term Drung (གྲོང་) serves as an honorific title in specific administrative and royal contexts. Considering that the lay figure is depicted with a halo, seated beneath a canopy, and addressed with this prestigious title alongside Shākya ‘od, his high status as a member of the royal family is highly probable. *The Royal Chronicles of Ngari* records that Shākya ‘od’s grandson was Blo bzang rab brtan [44]. Consequently, it is plausible that the lay figure represents Blo bzang rab brtan, who is shown as a figure venerated alongside his grandfather.

Supporting evidence is found in the donor murals of the *Lotsaba lha khang* in Riba Village, Zanda County, where a similar pair of figures is depicted. The inscription beneath the monk figure reads: *lha rje btsun Shākya ‘od kyi zhal snga nas*, meaning in the presence of the Divine Lord Venerable Master Shākya ‘od. The inscription beneath the lay figure reads: *lha mi dbang chen po drung Blo bzang (rab) brtan gyi zhal snga nas*, meaning *in the presence of the Great Sovereign of Gods and Men, the Honorable Blo bzang (rab) brtan*. Here, *lha* is a term reserved for descendants of the ancient Tibetan royal family, while *mi dbang chen po* clearly designates a king. This suggests that Blo bzang rab brtan may have already ascended the throne at the time of the Riba murals’ creation. In contrast, the use of the more modest title drung in the Mandala Hall suggests that he had not yet been enthroned when those murals were executed [45].

Historical records state that Blo bzang rab brtan was born in 1458 CE [43]. While no extant records document the exact year of his accession, the *autobiography of the Second Dalai Lama* records that in 1485 CE, Blo bzang rab brtan traveled to gTsang for an audience [46]. This indicates he had already ascended the throne of Guge by at least 1485 CE. In Yarlung tradition, the age of majority for royal succession was typically thirteen [43]; applying this custom to Guge, Blo bzang rab brtan would have been eligible to rule from 1471 CE onward.

Therefore, this study proposes that the Mandala Hall murals were completed between a TPQ of 1458 CE and a TAQ of 1471 CE; the VIF27 complex was likely constructed slightly earlier than the Mandala Hall.

#### 2.3.2. Textual evidence for the production date of the White Temple Buddha statues.

In 1980, a consecration prayer text (known as *zhuangzang fayuan wen* in Chinese) was discovered from the abdominal cavity of a Buddha statue at the site. The manuscript invokes divine blessings for King Jig rten dbang phyug pad dkar lde and his consort, offering prayers for their longevity, personal well-being, and the enduring prosperity of the Guge Kingdom [47]. This primary evidence is consistent with the view that the production occurred during the reign of King Jig rten dbang phyug. Historical records document his monastic patronage, including the construction of the White Temple and the Yamāntaka Hall at Tsaparang, which he undertook together with his brothers ‘Jam dbyangs pa and Chag mdo [40,42]. He presented precious gifts to the Second Dalai Lama in 1541 CE, established a monastic college for Ngari monks, maintained diplomatic contacts with the young Third Dalai Lama, and abdicated by 1555 CE [40,42,46]. This historical trajectory provides a conservative constraint that allows for strict chronological boundaries in our Bayesian model.

Therefore, Jig rten dbang phyug was primarily active during the mid-16th century. Accounting for the duration required for the production, ritual consecration, and final enshrinement of such monumental statuary, this study adopts a TAQ of 1560 CE as a conservative constraint—allowing a five‑year interval following his abdication. This historical constraint, derived independently of radiometric data, serves as a useful upper boundary for the subsequent Bayesian chronological modeling.

## 3. Results

### 3.1. Sample information

To establish a chronological framework for the construction history of the key structures of the ancient city of Guge, we collected and analyzed 12 plant specimens. The sample set comprises two primary material types: structural timber (n = 10) and short-lived botanical remains (n = 2), specifically herbaceous stalks. The inclusion of annual or short-lived species was a deliberate strategy to minimize the old-wood effect and ensure that the radiometric ages closely align with the actual construction or renovation.

Each specimen was documented *in situ* within its specific architectural layer or archaeological context. To prevent cross-contamination, all samples were sealed in dry containers prior to laboratory pretreatment. The detailed metadata for each specimen—including laboratory identification codes, material descriptions, and stable isotope (δ^13^C) values–is systematically summarized in Table 1.

#### 3.1.1. Samples from site VIF27.

Sampling Point 1 is located within the site designated VIF27 in *The site of the Ancient Guge Kingdom* [48]. The layout of VIF27 resembles the Chinese character “回” (hui). The center of the northern section of the inner wall projects outward to form a niche. Two wood samples (GAFA-2-No9 and GAFA-2-No10) were collected from this area. An additional wood sample (GAFA-2-No11) was taken from a timber embedded in the wall near the entrance (Fig 3). All three wood samples were obtained from the outermost layers of small-diameter timber (visually less than 10 cm in diameter) to minimize potential offsets from the old-wood effect. These embedded timbers are integral components of the original wall structure and bear no later cutting or replacement marks; furthermore, the building exhibits no visible evidence of subsequent repairs or structural additions.

**Fig 3:**
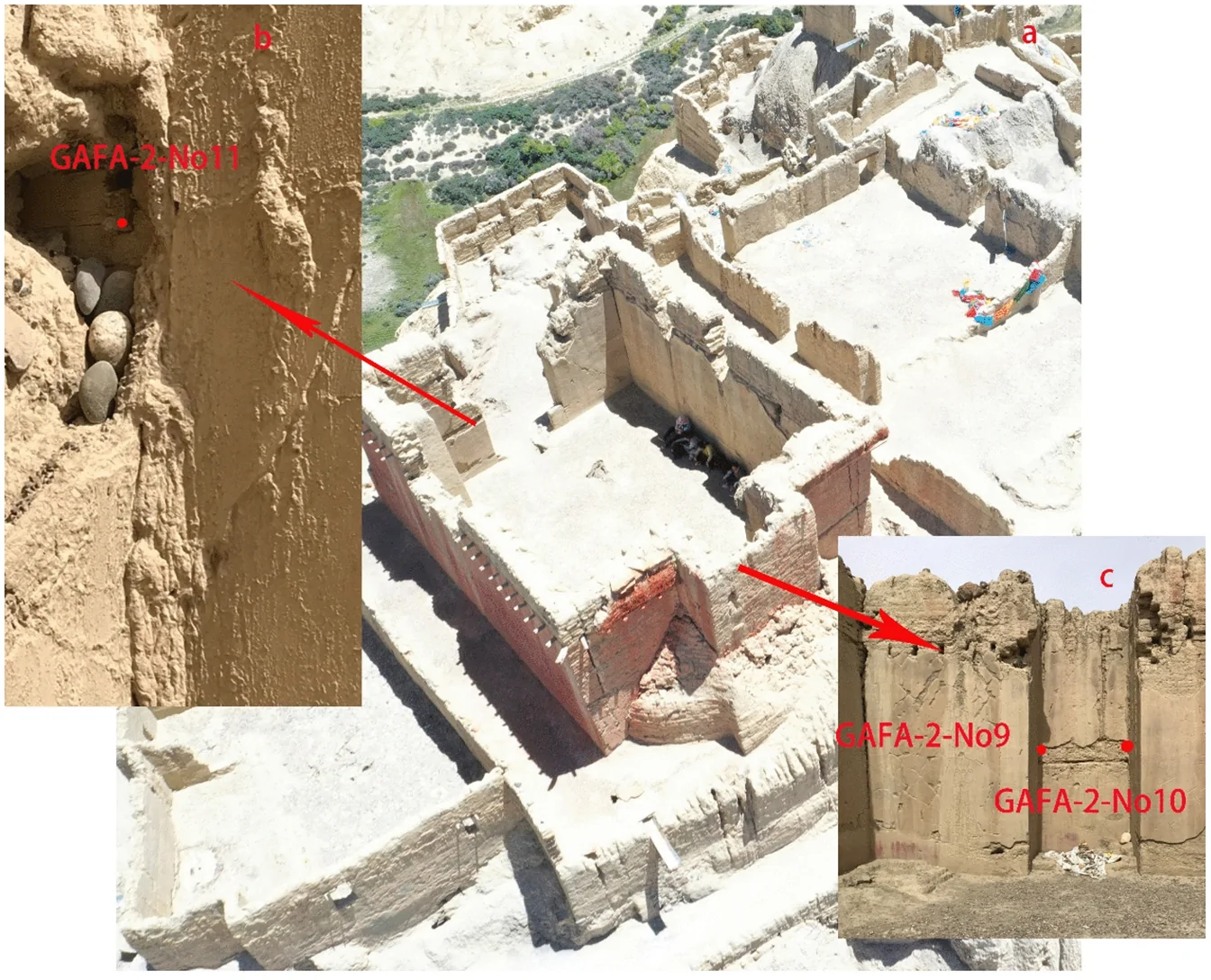
Sampling location at the VIF27 site. (a) Overview of the site; (b) Wooden rods in the niche wall (samples GAFA-2-No9 and No10); (c) Entrance opposite the niche (sample GAFA-2-No11) (photo by Jiaming Niu & Changqing Fu; CC BY 4.0).

#### 3.1.2. Buddha sculpture sample from site IVF262.

Sampling Point 2 is located within the site designated IVF262 in *The site of the Ancient Guge Kingdom* [48]. The northern wall once housed a clay sculpture; now, only the foundation of its pedestal and scattered wooden components remain. A wood sample (GAFA-2-No14) was collected from these fragmented wooden remains (Fig 4). The fragmented wood was found directly embedded within the original clay-plaster pedestal foundation; no later cutting, re-embedding, or replacement marks were observed, confirming that the sample dates to the primary sculptural construction phase.

**Fig 4:**
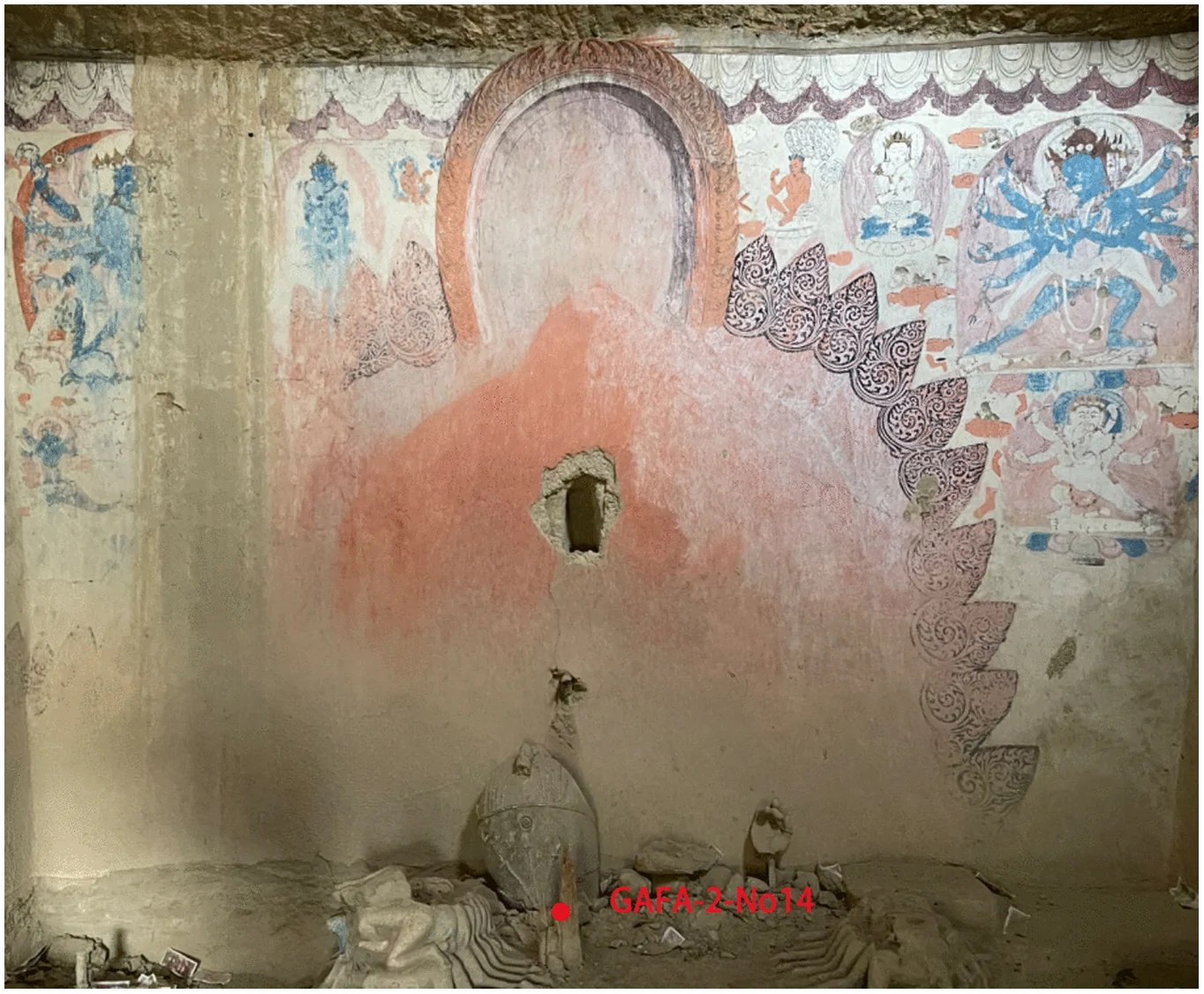
Sampling location at the IVF262 site (photo by Changqing Fu; CC BY 4.0).

#### 3.1.3. Buddha statue samples from white temple area O.

Sampling Point 3 is located within the White Temple (*Lha khang dkar po*), designated IVF189, in *The site of the Ancient Guge Kingdom* [48]. The site originally contained 23 statues, all of which are now damaged or incomplete; their original positions are shown in Fig 5a. At the northern end of the eastern wall altar, the first group of statues (designated Area O) retains only the lotus bases. Samples GAFA-2-No1, No2, No3, No5 are wood, while No4 and No6 are herbaceous stalks. Specifically, No1 was retrieved from a cruciform wooden armature; No2 and No3 were taken from wooden bases; No5 was collected from wood fragments scattered on the floor; and No4 and No6 were obtained from debris beneath the Buddha statue (Fig 5). All wooden and herbaceous stalk samples were retrieved from the original structural core of the statues (the cruciform armature, base, and debris beneath the pedestal). The absence of secondary repair layers or later reinstallation evidence strongly indicates that these samples belong to the initial production event.

**Fig 5:**
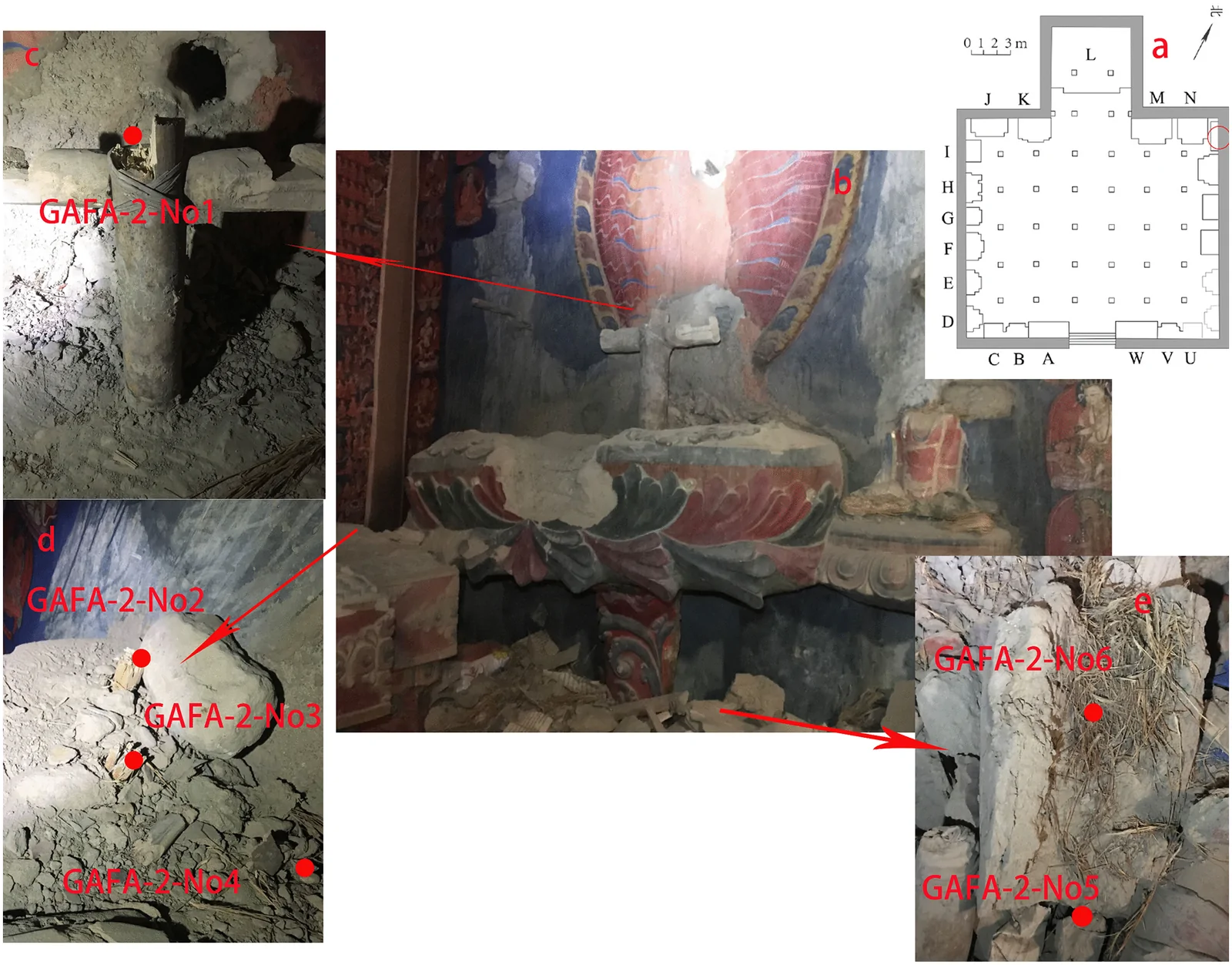
Sampling location at the White Temple Area O (photo by Jiaming Niu; CC BY 4.0). (a) Location diagram of Area O; (b) Overview of the site; (c) the central area of the cross-shaped wooden skeleton (GAFA-2-No1); (d) wooden base on the right (GAFA-2-No2 and No3), herbaceous stalks sample collected near the base (GAFA-2-No4); (e) scattered wooden fragments on the ground (GAFA-2-No5); the statue debris on the ground (GAFA-2-No6).

#### 3.1.4. Watchtower samples from site VIII D2.

Sampling Point 4 is located within the watchtower designated VIII D2 in *The site of the Ancient Guge Kingdom* [48]. This structure features a circular floor plan, and its encircling wall is pierced with narrow, slit-shaped, or triangular arrow loops. Two wood samples (GAFA-2-No7 and No8) were collected from exposed timbers (Fig 6). Both samples were obtained from the outermost layers of small-diameter wood (visually less than 2 cm in diameter). These timbers are integral to the original masonry and show no signs of being later replacements, thereby providing reliable material for dating the primary construction of the watchtower. The sampled twigs were incorporated into the original mud-brick masonry that forms the arrow-slit supports; their sealing by undisturbed mortar and the lack of any later cutting or replacement features demonstrate that they represent the primary fortification phase rather than a subsequent repair.

**Fig 6:**
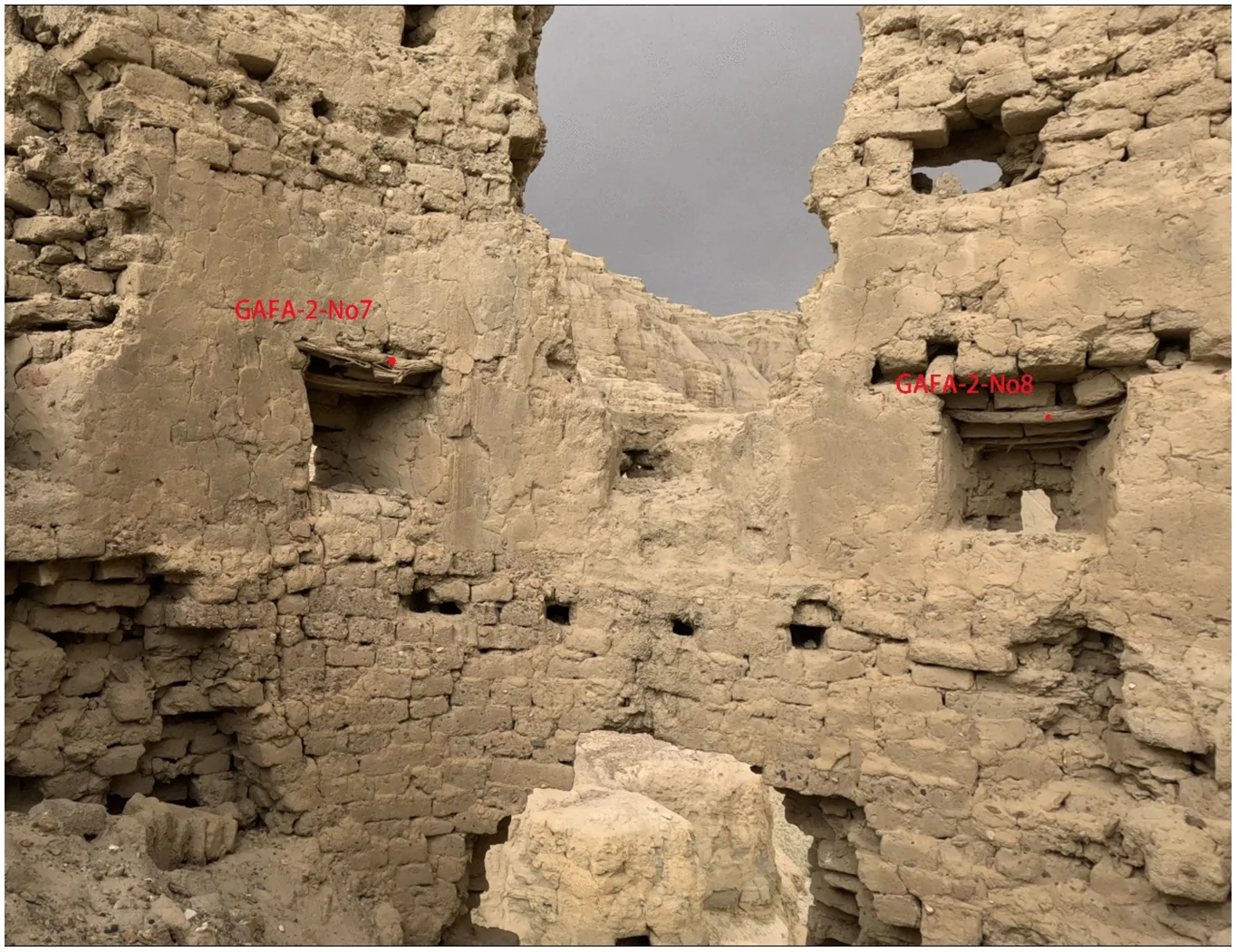
Sampling location at the VIII D2 site (photo by Jiaming Niu; CC BY 4.0).

### 3.2. Radiocarbon and Bayesian chronological results for the sampled structures

#### 3.2.1. Construction date of the VIF27 complex.

When the three wood samples were combined using OxCal’s *Combine* command within a Phase structure, they yielded an agreement index of *A*_comb_ = 111.1, exceeding the critical threshold and confirming their statistical consistency as a single construction event.

In the unconstrained model, the combined samples produce a broad 95.4% Highest Posterior Density (HPD) interval of 1456–1616 CE (Fig 7a). This wide range results from wiggles in the IntCal20 calibration curve and includes a secondary probability peak after 1560 CE, introducing chronological ambiguity.

**Fig 7:**
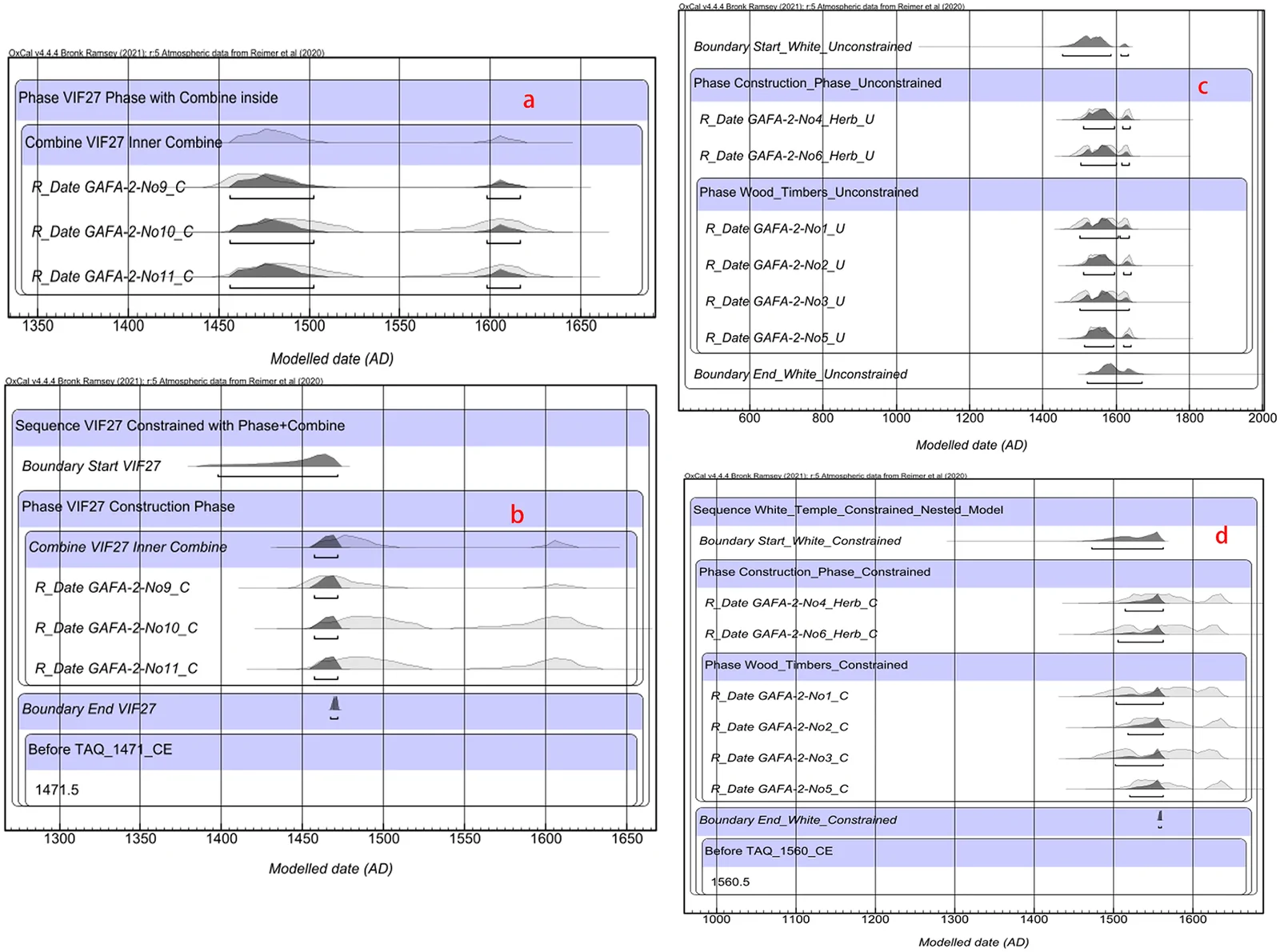
Bayesian modeled posterior probability distributions for the VIF27 complex and the White Temple statue (CC BY 4.0). (a) Unconstrained model for the VIF27 complex; (b) Constrained model for the VIF27 complex with a TAQ of 1471 CE; (c) Unconstrained nested model with herb and wood sub-phases for the White Temple statues; (d) Constrained nested model for the White Temple statues with a TAQ of 1560 CE.

To resolve this multimodality, the TAQ of 1471 CE (see Section 2.3.1) was applied as a Before condition in a constrained sequence. The constrained Phase+*Combine* model eliminates the post‑1560 CE peak entirely and yields a posterior 95.4% HPD interval of 1457–1471 CE for the combined construction event (Fig 7b). The model also estimates the end of the construction phase (Boundary End) at 1467–1471 CE (95.4% HPD), indicating that while the primary timber use occurred between 1457 and 1471 CE, the final completion of the building fell within the narrower window of 1467–1471 CE. The overall model performance is excellent (*A*_model_ = 99.2, *A*_overall_ = 98.4; see Supporting Information S1 File–S2 File for OxCal code). These results demonstrate how an independent historical prior can objectively resolve calibration‑curve ambiguities and constrain both the construction event and the completion boundary of the VIF27 complex.

#### 3.2.2. Production date of the Buddha statue at IVF262.

Sample GAFA-2-No14 yielded a ^14^C age of 405 ± 20 BP (Table 1). Calibration using the IntCal20 curve yields two distinct calendar age ranges at the 95.4% confidence level: 1442–1501 CE (88.9% probability) and 1599–1616 CE (6.6% probability). While the earlier range has a substantially higher probability, the later range cannot be dismissed on statistical grounds alone. The full calibration results are presented in Table 1; the historical interpretation of these two ranges is provided in Section 4.4.

#### 3.2.3. Production date of the White Temple Area O Buddha statues.

Using an unconstrained nested Bayesian model (two sub-phases: herbs and wood), we find that the unmodeled calibrated 95.4% HPD intervals for the six samples range from 1479 to 1649 CE, while the modeled posterior intervals narrow to 1500–1640 CE. The unconstrained production phase boundaries span from 1454 CE (start) to 1668 CE (end) at the 95.4% HPD level.

Applying a TAQ of 1560 CE as a boundary constraint (see Section 2.3.2 for the derivation of this TAQ) forces the end of the production event to lie before 1560 CE. In the constrained nested model, the posterior 95.4% HPD interval for the end boundary is 1556–1560 CE, and the individual sample posterior ranges narrow to 1502–1563 CE (with all wooden samples having an upper bound of 1563 CE and the herb samples showing slightly earlier lower bounds; see Supporting Information S3 File–S4 File). The model exhibits excellent statistical coherence: the unconstrained nested model yields *A*_model_ = 107.5 and *A*_overall_ = 108.1; the constrained model’s end boundary has an agreement index A = 100, satisfying the TAQ prior (Fig 7c-7d).

#### 3.2.4. Construction date of watchtower VIII D2.

Both wood samples from the VIII D2 watchtower consist of small twigs characterized by few growth rings and a near-absence of heartwood. These attributes reduce the potential for the old-wood effect and reuse; consequently, the dating results are likely to reflect the construction or reinforcement of the watchtower. The two samples yielded ^14^C ages of 177 ± 20 BP and 171 ± 20 BP, respectively. Calibration using the IntCal20 curve provides three calendar age ranges at 95.4% confidence level: 1661–1695 CE, 1725–1813 CE, and a marginal low-probability interval in the 19th–20th centuries (Table 1).

Owing to fluctuations in the calibration curve in this segment, the radiocarbon data alone cannot statistically distinguish between the 1661–1695 CE and 1725–1813 CE intervals; the two intervals are statistically indistinguishable, and neither can be preferred over the other on radiometric grounds. All calibrated ranges are presented in Table 1; their historical interpretation is provided in Section 4.4.

All OxCal code, posterior probability plots, agreement indices, and comparisons between constrained and unconstrained models for VIF27 and White Temple statues are provided in Supporting Information.

## 4. Discussion

The chronological interpretations presented below are based on probabilistic calibration and Bayesian modeling; as such, all age estimates carry inherent uncertainty. Where single-sample contexts or multimodal calibration distributions occur, we explicitly acknowledge alternative possibilities and avoid overstatement. With this caveat, we proceed to interpret the results in their historical and architectural context.

### 4.1. Assessment of the old wood effect

The old-wood effect is a recognized source of systematic uncertainty in archaeological radiocarbon dating. Although we preferentially sampled the outer portions of timbers and herbaceous stalks (GAFA-2-No4 and No6) during fieldwork, the potential offset remains a factor to be considered. Exact wood species could not be identified; the estimated offsets below are conservative upper limits based on general ranges reported in archaeological radiocarbon dating guidelines.

VIF27 site. The three wood samples were taken from outer portions of small-diameter timbers (visually estimated <10 cm). Based on typical old-wood offsets for such samples, we assume a conservative maximum offset of ≤50 years for each sample. Even if a full 50-year offset were applied to the raw ^14^C ages before modeling, the resulting construction date would still fall within the late 15th to early 16th centuries, which does not contradict the historical constraints.

IVF262 Buddha statue. The sample (GAFA-2-No14) was taken from a small fragment of the outer portion of a timber after the bark had been removed. We assume a conservative maximum offset of ≤20 years. Therefore, the calibrated earlier range (1442–1501 CE) is likely accurate to within two decades, placing the most probable production date of the statue in the late 15th century.

White Temple Buddha statues (Area O). Six samples were analyzed, including two herbaceous stalks (GAFA-2-No4 and No6) with negligible old-wood offset (≤1 year), providing a firm TPQ for the statue production event. The four wooden samples are small fragments taken from outer portions; we assume a maximum offset of ≤30 years. Incorporating the historical constraint (TAQ 1560 CE) in a nested Bayesian model that separates herb and wood samples produces posterior 95.4% HPD ranges of 1502–1563 CE for the individual statue components, with the end boundary of the construction event constrained to 1556–1560 CE (see Section 4.4.3). Even if a maximum plausible old‑wood offset of 30 years were applied to the wooden samples, the actual production date would still fall within the 16th century and remain compatible with the historical records.

Watchtower VIII D2. The two samples (GAFA-2-No7 and No8) were taken from small branches or twigs incorporated into the watchtower structure. Twigs consist almost entirely of recent growth (often less than 5–10 years), so the old-wood effect is minimal. We assume a maximum offset of ≤2 years. Therefore, the calibrated ^14^C ages are close to the actual felling date, and the multimodal calibration distribution (95.4% probability of 1661–1813 CE, with primary intervals 1661–1695 CE and 1725–1813 CE) is primarily a function of the IntCal20 curve’s fluctuations, not an artifact of old wood. The construction or reinforcement of the watchtower is most likely to have occurred within the late 17th or 18th century, consistent with documented periods of conflict or administrative change (Section 3.2.4). The possibility of significant old wood offset or reuse is minimal due to the use of small branches.

Limitations and future directions. The absence of wood species identification and tree-ring analysis limits the precision of our old-wood corrections. A regional dendrochronological reference for western Tibet would be invaluable for refining these estimates. Owing to the lack of species identification, the assumed maximum offsets (ranging from ≤20 to ≤50 years depending on the context) are conservative estimates based on archaeological guidelines for temperate regions; for long-lived conifers, the actual offset could be larger. These offset values are heuristic estimates rather than empirically derived figures because no regional dendrochronological or wood-anatomical data are currently available. Future work should prioritize species identification and tree-ring analysis, and take multiple samples from individual timbers where possible to assess intra-tree variability. Despite these limitations, the consistency between the ^14^C results (after accounting for plausible old-wood offsets) and the independent historical constraints (e.g., the TAQ of 1471 CE, the reign of Jig rten dbang phyug) supports the overall robustness of the proposed chronological framework.

### 4.2. Evidence for single phase construction

For the structures sampled in this study, we took the following steps to assess the possibility of intra-building multi-phase construction:

Internal consistency of ^14^C dates. When the three VIF27 samples were combined using OxCal’s *Combine* command within a Phase structure, an agreement index of *A*_comb_ = 111.1 was obtained. When placed in a Bayesian sequence model with the TAQ constraint, the combined samples produced a tight posterior range (1457–1471 CE). If later repairs had occurred, some samples would be expected to be significantly younger, but this was not observed.

Similarly, the six White Temple samples were analyzed in a nested Bayesian model (two sub-phases for herbs and wood) without forcing a common age. The unconstrained model achieved high overall agreement indices (*A*_model_ = 107.5, _*A*overall_ = 108.1). In the constrained model (TAQ 1560 CE), all individual sample agreement indices (A) exceed 79, and the end boundary has A = 100, confirming that all six samples are statistically consistent and represent a single production event.

Watchtower VIII D2. The use of small branches or twigs—which are unlikely to have been reused from older structures or to have survived as later replacements—argues against multi-phase construction. The two samples are statistically consistent, and their calibrated ranges overlap entirely.

Therefore, we conclude that each sampled structure represents a single, discrete construction or production event, rather than a multi-phase sequence. The three major phases identified in Section 4.4 correspond to different buildings or different parts of the site, not to successive renovations of the same building.

Nevertheless, we acknowledge that our sample set is limited (12 samples from four areas) and cannot definitively rule out the presence of later minor repairs that left no recognizable traces or were not sampled. Future work should adopt a more intensive sampling strategy within individual buildings—for example, collecting samples separately from foundations, wall cores, and upper timbers—and combine them with Bayesian sequence modeling to detect potential construction phases.

### 4.3. Critical evaluation of Tibetan historical sources

The chronological arguments in this study rely on several Tibetan historical texts, including *the Royal Chronicles of Ngari*, Sum pa Ye shes dpal ‘byor’s *History of Buddhism*, Desi Sanggye Gyatso’s *Yellow Beryl*, and the Fifth Dalai Lama’s *Biographies of the First to Fourth Dalai Lamas*. A critical assessment of these sources is necessary because Tibetan historiography differs from the empirical and archival traditions of European historiography.

First, oral transmission and late codification. Systematic historical writing in Tibet emerged only after the introduction of Buddhism in the 7th–9th centuries; earlier events often relied on oral traditions committed to writing centuries later.

Second, the religious framing of historical narrative. Tibetan historical works are composed within a Buddhist worldview, where the narration of events often serves religious didactic purposes rather than strict chronological accuracy. The authors of the texts cited in this study—including Sum pa Ye shes dpal ‘byor (1704–1788), a prominent Gelug scholar, and Desi Sanggye Gyatso (1653–1705), a high-ranking religious and political figure—wrote from within the Buddhist historiographical tradition. Their accounts of earlier periods (e.g., 15th–16th-century Guge) are not contemporaneous records but retrospective compilations based on earlier sources that are no longer extant.

Third, textual variants among manuscript traditions. Tibetan manuscripts often exhibit variant readings in different recensions. For example, the death of the last Tibetan emperor, Glang Darma, is recorded in different sources as 842 CE and 846 CE, illustrating that even key dates can vary across manuscript traditions. While we have not identified contradictory variants for the specific dates used in this study, the possibility of such variants cannot be ruled out.

Despite the aforementioned limitations, relatively reliable chronological deductions can be achieved for several pivotal events:

Concerning late 15th century events, the key dates—King Phun tshogs lde’s monastic ordination in 1449 CE, the birth of King Blo bzang rab brtan in 1458 CE, and Phun tshogs lde’s death in Guge in 1481 CE—are all derived from *The Royal Chronicles of Ngari*. Although this work is not a strictly contemporary administrative record of late 15th-century events, its author, Ngag dbang grags pa, held a special position: as early as 1424, he attended the wedding and coronation of King Phun tshogs lde, served as the abbot of Tholing Monastery, was the supreme master in Guge, and lived through the reigns of five kings (rNam rgyal lde, Phun tshogs lde, rNam rgyal sangs rgyas lde, Blo bzang rab brtan, and ‘Phags pa lha). Given his personal experience and high status, The Royal Chronicle of Ngari, as a “near-contemporary” compiled work, possesses high credibility in its accounts of the period from Phun tshogs lde onward [43].

Concerning mid 16th century events: The historical accounts of King Jig rten dbang phyug’s audiences with the Second Dalai Lama (1541 CE) and the Third Dalai Lama (1546 and 1555 CE) yield an even higher degree of chronological resolution. The 1541 CE audience is documented directly in the Autobiography of the Second Dalai Lama—a firsthand, eyewitness record penned by the subject himself. The audiences of 1546 CE and 1555 CE are documented in the *Autobiography of the Third Dalai Lama*. The latter, although compiled under the direction of the Fifth Dalai Lama, draws on primary contemporary materials (including letters and official documents), and therefore offers a higher degree of chronological accuracy than retrospective chronicles.

It should be noted that the majority of the chronological data utilized in this study do not stem from strictly administrative or lapidary archives. The potential existence of divergent chronological traditions within other unexamined textual lineages, though currently undetected, cannot be completely dismissed. In this study, we have adopted the dates derived from these texts as the best available historical constraints, while explicitly acknowledging the limitations inherent in their retrospective and religiously framed nature. Where possible, we have cross referenced multiple sources (e.g., the Mandala Hall inscriptions, the *Lotsaba Lhakhang* murals) to triangulate the chronology. The radiocarbon dates provide an independent check on the textual evidence, and the close agreement between the two lines of evidence increases confidence in the overall chronological framework, despite the uncertainties inherent in each individual source. Future discovery of contemporaneous administrative documents or inscriptions may further refine these dates.

### 4.4. Three construction phases and their historical significance

This study proposes a refined chronological framework for the architectural evolution of the Guge ancient city by integrating AMS ^14^C dating, Bayesian statistical modeling, and historical-epigraphic analysis. Based on the convergence of radiometric data and historical priors, we define three distinct construction phases that characterize the site’s development.

#### 4.4.1. Early Construction Phase (mid-to-late 15th century).

This period encompasses Site VIF27, the Mandala Hall, and Site IVF262. The Bayesian model for VIF27 produced a posterior date of 1457–1471 CE. Importantly, this aligns closely with the chronology of the Mandala Hall (1458–1471 CE), which was independently inferred from mural inscriptions. As detailed in Section 2.3.1, the TAQ of 1471 CE for the VIF27 complex is derived from the Mandala Hall murals. The three wood samples from VIF27 are statistically consistent, and their combined posterior range falls within the mid‑15th century, consistent with the reigns of King Phun tshogs lde and his successor.

Concurrently, the Buddha statue in IVF262 is most likely from 1442–1501 CE. The dating of the IVF262 Buddha statue warrants additional discussion due to its bimodal calibration result. The earlier range (1442–1501 CE) coincides with the reigns of King Phun tshogs lde and his son rNam rgyal sangs rgyas lde, a period of prolific royal Buddhist patronage and systematic expansion of early religious sites. The later range (1599–1616 CE) would place the statue in the early 17th century, immediately before the Ladakhi conquest of Guge (c. 1630 CE), a period during which large-scale, royally sponsored construction projects appear notably absent from historical records due to increasing internal and external conflicts. Although the later range cannot be definitively excluded, the convergence of independent evidence (historical context, and the higher probability density of the earlier range) favors a 15th-century attribution. Future systematic sampling from secure stratigraphic contexts would help resolve this ambiguity.

#### 4.4.2. White Temple Phase (mid-16th century).

The White Temple is a major religious structure within the Tsaparang complex. As detailed in Section 2.3.2, historical records confirm that King Jig rten dbang phyug, together with his brothers, founded the White Temple and the Yamāntaka Hall at Tsaparang [40,42].

The Buddha statues in Area O of the White Temple were most likely produced during a construction event whose end is tightly constrained to 1556–1560 CE (95.4% HPD), with the felling dates of the structural timber and the harvest dates of the herbaceous stalks ranging from approximately 1502–1563 CE. This interval coincides with the reign of King Jig rten dbang phyug and the years immediately following his abdication, in full agreement with the historical TAQ of 1560 CE (see Section 2.3.2). The short-lived herb samples (GAFA-2-No4 and No6) provided precise temporal anchors with negligible old-wood offset, and when integrated with the TAQ constraint, their modeled posterior ranges (1506–1563 CE and 1516–1563 CE, respectively) effectively resolved the chronological ambiguity typically induced by the 16th-century calibration plateau. The White Temple could also have been built during this period.

#### 4.4.3. Late Fortifications (mid-17th to late 18th century).

Regarding the watchtower at Site VIII D2, its construction or major structural reinforcement likely falls within the period between the mid-17th and late 18th centuries. Although the 19th–20th–century range appears as a statistical possibility in the calibration output, it is inconsistent with the observed degree of structural weathering and the morphological characteristics of the surrounding architectural remains. Given the lack of archaeological support, it should not be adopted as a reliable construction date.

Two possible historical contexts may account for the watchtower’s construction or reinforcement, but the radiocarbon data alone do not allow a definitive choice between them. The 1661–1695 CE interval coincides with the final phase of Ladakhi rule over Guge and the Tibet–Ladakh War (1679–1684 CE). Fortification during this period could represent defensive efforts by Ladakhi occupation forces or post-war reconstruction under the newly established Tsaparang Dzong administration. The 1725–1813 CE interval falls within a period when Western Tibet faced multiple external threats, including sustained Dzungar pressure in the 1720s and the Gorkha invasions of 1788 and 1791 CE, which may have prompted the local Tibetan government to fortify the Ngari frontier and re-establish Tsaparang as a military stronghold. Both scenarios remain plausible, and the evidence currently available does not permit a definitive selection.

These three phases illustrate the negative dialectic between *tabula rasa* and *palimpsest* in Guge’s urban evolution [49]. The initial phase exhibits a dual character: the remodeling of 10th–11th centuries vihara-style monasteries and the construction of the newly planned VIF27 temple complex (*tabula rasa*-style innovation). The 16th-century royal patronage in the White Temple reinforces historical accumulation, whereas the late-stage fortifications represent functional adaptations to external existential threats. This dialectical relationship between rupture and continuity constitutes the deep-seated logic through which the Guge site negotiates its own history. **The broader theme of historical continuity and transformation resonates across disciplinary boundaries, from studies of genetic heritage in population history [**50**] to the measurement of intangible cultural heritage [**51].

## 5. Conclusion

This study presents the first high-resolution construction chronology for key structures of the Guge ancient city by integrating AMS ^14^C dating, Bayesian modeling, and textual and epigraphic analysis. Three major phases are identified: mid-to-late 15th century (VIF27, Mandala Hall, IVF262), mid 16th century (White Temple statues under King Jig rten dbang phyug), and mid 17th to late 18th century (Watchtower VIII D2). The use of short-lived plant remains successfully resolved calibration plateaus, and the consistency between radiocarbon results and independent historical constraints demonstrates the robustness of the framework. While our framework is robust for structures with multiple samples (VIF27, White Temple), some chronological ambiguities remain—particularly for single-sample contexts such as IVF262 and for watchtower VIII D2 where calibration curve fluctuations produce two equally plausible intervals. These uncertainties underscore the value of future systematic sampling and regional dendrochronological development. The findings provide a scientific basis for understanding the architectural and socio-political evolution of the Guge Kingdom, and future work should expand sampling across more structures to further refine the chronology.

## Supporting information

S1 File OxCal code for VIF 27. (DOCX)

S2 File OxCal output CSV for VIF27. (CSV)

S3 File OxCal code for White temple Statue. (DOCX)

S4 File OxCal output CSV for White Temple Statue. (CSV)
