# Supplementary material for: Dating the key structures of the Guge ancient city through integrated 14C and literary research
Source: PLoS One. 2026 Sep 30;21(9):e0353853. doi: 10.1371/journal.pone.0353853 (PMC13626318; doi:10.1371/journal.pone.0353853)
Supplement: Supplementary file 1 [file pone.0353853.s001.docx]

## **S1 OxCal code for VIF 27**

**1. Model A:** **Unconstrained model (no TAQ)**

Plot()

{

Phase("VIF27 Phase with Combine inside")

{

Combine("VIF27 Inner Combine")

{

R_Date("GAFA-2-No9_C", 398, 20);

R_Date("GAFA-2-No10_C", 367, 20);

R_Date("GAFA-2-No11_C", 373, 20);

};

};

};

**2. Model B: Constrained Bayesian Sequence Model with TAQ 1471 CE**

Plot()

{

Sequence("VIF27 Constrained with Phase+Combine")

{

Boundary("Start VIF27");

Phase("VIF27 Construction Phase")

{

Combine("VIF27 Inner Combine")

{

R_Date("GAFA-2-No9_C", 398, 20);

R_Date("GAFA-2-No10_C", 367, 20);

R_Date("GAFA-2-No11_C", 373, 20);

};

};

Boundary("End VIF27")

{

Before("TAQ_1471_CE", AD(1471));

};

};

};
