# Supplementary material for: Dating the key structures of the Guge ancient city through integrated 14C and literary research
Source: PLoS One. 2026 Sep 30;21(9):e0353853. doi: 10.1371/journal.pone.0353853 (PMC13626318; doi:10.1371/journal.pone.0353853)
Supplement: Supplementary file 2 [file pone.0353853.s003.docx]

## **S3 OxCal code for White temple Statue**

**1. Model A: Unconstrained Nested Phase Model**

Plot()

{

Sequence("White_Temple_Unconstrained_Nested_Model")

{

Boundary("Start_White_Unconstrained");

Phase("Construction_Phase_Unconstrained")

{

R_Date("GAFA-2-No4_Herb_U", 314, 20);

R_Date("GAFA-2-No6_Herb_U", 332, 20);

Phase("Wood_Timbers_Unconstrained")

{

R_Date("GAFA-2-No1_U", 337, 20);

R_Date("GAFA-2-No2_U", 309, 20);

R_Date("GAFA-2-No3_U", 340, 20);

R_Date("GAFA-2-No5_U", 304, 20);

};

};

Boundary("End_White_Unconstrained");

};

};

**2. Model B: Constrained nested model with TAQ 1560 CE**

Plot()

{

Sequence("White_Temple_Constrained_Nested_Model")

{

Boundary("Start_White_Constrained");

Phase("Construction_Phase_Constrained")

{

R_Date("GAFA-2-No4_Herb_C", 314, 20);

R_Date("GAFA-2-No6_Herb_C", 332, 20);

Phase("Wood_Timbers_Constrained")

{

R_Date("GAFA-2-No1_C", 337, 20);

R_Date("GAFA-2-No2_C", 309, 20);

R_Date("GAFA-2-No3_C", 340, 20);

R_Date("GAFA-2-No5_C", 304, 20);

};

};

Boundary("End_White_Constrained")

{

Before("TAQ_1560_CE", AD(1560));

};

};

};
